# Effects of a systemic enzyme therapy in healthy active adults after exhaustive eccentric exercise: a randomised, two-stage, double-blinded, placebo-controlled trial

**DOI:** 10.1136/bmjsem-2016-000191

**Published:** 2017-03-12

**Authors:** Tobias Marzin, Gerhard Lorkowski, Claudia Reule, Stefanie Rau, Elisabeth Pabst, Johannes C Vester, Helmut Pabst

**Affiliations:** 1 Zentrum für Leistungsdiagnostik, Sportschule Fuerstenfeldbruck-Puch Gmbh, Fuerstenfeldbruck, Germany; 2 GL Pharma Consulting Research & Development, Gauting, Germany; 3 BioTeSys GmbH, Esslingen, Germany; 4 Mucos Pharma GmbH & Co. KG, Unterhaching, Germany; 5 Sportmedizin, Gilching, Germany; 6 IDV Data Analysis and Study Planning, Krailling, Germany

**Keywords:** Muscle injury and inflammation, exercise, recovery, proteolytic enzymes

## Abstract

**Background:**

Systemic enzyme therapy may improve symptoms of exhaustive eccentric exercise due to anti-inflammatory properties.

**Methods:**

In a randomised, placebo-controlled, two-stage clinical trial, systemic enzyme therapy (Wobenzym) was administered for 72 hours before and 72 hours following a day on which subjects performed an exhaustive eccentric exercise (isokinetic loading of the quadriceps). Efficacy criteria (maximal strength and pain) and time points were selected to account for the multidimensional nature of exercise-induced muscle damage symptoms. Subjects were randomised in a crossover (stage I, n=28) and parallel group design (stage II, n=44).

**Results:**

Analysis of stage I data demonstrated a significant superiority (Mann-Whitney=0.6153; p=0.0332; one sided) for systemic enzyme therapy compared with placebo. Stage II was designed as a randomised controlled parallel group comparison. Heterogeneity (I^2^>0.5) between stages led to separate analyses of stage I (endurance-trained subjects) and stage II (strength-trained subjects). Combined analysis resulted in no evidence for corresponding treatment effects. Analysis of pooled biomarker data, however, demonstrated significant favourable effects for systemic enzyme therapy in both stages.

**Conclusion:**

Systemic enzyme therapy before and after exhaustive eccentric exercise resulted in higher maximal concentric strength in the less strength-trained subjects (stage I) and in significant favourable effects on biomarkers (inflammatory, metabolic and immune) in all subjects. The application of these findings needs further evaluation.

**What are the new findings?**

This study confirms a substantial and significant effect of systemic enzyme therapy (SET) on fatigue, muscle soreness and damage, as well as immunological and metabolic biomarkers, in male sportsmen with medium performance level (mostly runners and general athletes). Muscle soreness and maximal strength were not improved in those subjects with a higher level of strength training at baseline.
Use of SET showed a significant reduction in inflammatory biomarkers in sportsmen across all training levels, indicating an application for SET in supporting normal inflammatory processes for muscle recovery.
Clinicians may recommend the use of SET for mediating muscle fatigue, reducing soreness and attenuating potential muscle damage in endurance athletes.

## Introduction

Exercise-induced muscle damage (EIMD) and its most common symptom, delayed onset of muscle soreness (DOMS), impact an athlete’s training frequency and performance. Strenuous exercise, acute or postsurgical trauma and certain diseases can all be sources of skeletal muscle injury. Regardless of the type of injury, the general injury and repair mechanism are similar^1 2^ and have been well characterised in EIMD.^3–11^

Inflammation contributes to fibrosis^12^ and causes pain that may impair skeletal muscle function.^11^ Therefore, it has been common practice to reduce inflammation with drugs, such as COX-2 inhibitors. The problem with this approach is that while inflammation causes further injury to muscles,^1 2 13 14^ preventing inflammation may hinder recovery.^1 15–17^ As a result, current treatment options for inflammation are not necessarily effective and, in some cases, they may be unsafe.

Systemic enzyme therapy (SET)^i^ allows inflammatory processes to progress naturally and this overcomes the problem of preventing inflammation in a manner that may hinder recovery. The antioxidant rutin reduces oxidative stress during inflammation.^18 19^ Orally administered proteolytic enzymes, also called proteinases, are mainly absorbed in the small intestine and are active in body fluids and tissues as free and bound proteinases, despite their low concentrations (pmol–nmol).^20^ Trypsin and bromelain share two main biological activities with other proteinases: (i) they degrade proteins by their proteolytic activity which cleaves peptide bonds at specific sites, both in digestion and as markers of cell destruction and inflammation, and (ii) they bind to specific (eg, α_2_-antitrypsin) or unspecific (eg, α_2_-macroglobulin) antiproteinases to prevent uncontrolled protein degradation.

Accumulating evidence points to a role of protease-activated receptor 2, expressed on T cells, eosinophils, neutrophils and mast cells, in the regulation of inflammation and immune function.^20–23^ Further, both trypsin and bromelain form complexes with α_2_-macroglobulin leading to a conformational change that exposes receptor recognition sites in each of its four subunits.^20 24^ These complexes are recognised by low-density lipoprotein receptor-related protein and cell surface glucose-regulated protein (GRP78) receptors^24^ on blood and immune cell surfaces resulting in modification of cellular activities^25^ and rapid elimination by hepatocytes.^20 26^ During inflammation the complex of protease and antiprotease is subject to further modifications.^20^ Oxidation of the proteinase antiproteinase complex may serve as a switch mechanism that downregulates the progression of acute inflammation by sequestering TNF-α, interleukin (IL) 2 and IL-6, while upregulating the development of tissue repair processes by releasing bFGF, b-NGF, PDGF and TGF-β.^20^ Thus, SET may affect EIMD and DOMS by balancing the inflammatory response to injury.

The influence of oral proteinases on EIMD was investigated in several clinical trials focusing on pain and/or muscle function and strength.^27–29^ Bromelain and other proteinases may reduce muscle inflammation after EIMD,^1^ but controversy persists.^27 28^ The aim of the current trial was to investigate the effects of SET before and after exhaustive eccentric exercise on functional and biochemical parameters of EIMD and DOMS in male sportsmen who had a medium level of performance.

## Methods

### Trial design

This was designed as a prospective, randomised, double-blinded, placebo-controlled, two-stage trial. Stage I was a crossover with a washout phase of 21 days between phases; stage II was continued as parallel group comparison. Subjects were randomised to either SET or placebo (stage I: n=2×14, crossover; stage II: n=2×22, parallel group) and involved parties were blinded, except for the person responsible for interim (stage I) and final confirmatory analyses. Administration of medication began 72 hours before an exhaustive eccentric exercise day and continued for 72 hours following the muscle damaging exercise. Each subject completed an activity diary and a food frequency questionnaire. The clinical trial complied with the Declaration of Helsinki and was approved by the local health authority and ethical committee. It was registered at ClinicalTrials.gov (NCT01845558).

### Subjects

Subjects were aged 20–50 years, had a body mass index between ≥20 kg/m^2^ and ≤32 kg/m^2^ and had a moderate performance level and moderate strength ability defined by a medium concentric strength ability of 150–300 Nm (newton metre) peak torque maximum (PTM). Nutritional status was assessed via questionnaire at screening; subjects were asked not to change nutritional habits; and standardised meals were offered during the exercise day. Subjects were not to practise any physical activity (including driving to work by bicycle) during the study. All physical activities were documented. Subjects were also instructed to avoid activities after the exercise such as massages or hot bathing or showering. Relevant exclusion criteria were history or presence of any medical disorder, intake of anti-inflammatory medication, food supplements or use of other procedures directly affecting muscle function or performance within 4 weeks prior to or during trial. Intake of analgesic medication or consumption of alcohol was not allowed 24 hours prior and until 72 hours after exercise.

### Sample size

Non-parametric sample size calculation within the framework of a multiple outcome approach^30–33^ was performed applying the validated software Nnpar V.1.0 (IDV, Gauting, Germany). With the sample size of 30 subjects, the power calculation for stage I indicated a 64% chance (power of 90%) to detect a ‘medium-sized’ group difference with respect to the multidimensional test. If neither success nor futility was formally determined after stage I, a subsequent stage II could be planned based on the results of stage I (sample size reassessment with adaptive design features).^34 35^ Recalculated total sample size for stage II was 2×22 subjects (one phase, no crossover design).

### Interventions

Subjects were recruited through a telephone questionnaire and invited to the screening visit 2–4 weeks before exercise to give informed consent and confirm eligibility. Demographic data, medical history, physical activity (Freiburger Activity Questionnaire), nutritional performance status, vital signs and blood samples for routine parameters were collected.

After a 5 min warm-up period on an ergometer, the maximal concentric strength of the quadriceps femoris muscle of the stronger leg, determined at screening, was measured on a desmodrom (Fa. Schnell). Three repetitions of 20 maximal concentric contractions were performed (60 s at 20 oscillations per minute) with 1 min passive recovery between sets. The highest value was used for analysis. For each measurement, peak torque and angle of peak torque were documented. On day 4 of the trial (visit 1), EIMD was induced by exhaustive eccentric exercise according to McLeay.^36^ Maximal eccentric, isokinetic loading of the right and left quadriceps femoris was determined by using a desmodrom (Fa. Schnell) as before, but with the difference of eccentric, not concentric, exercise. The same observer motivated all subjects following a standardised protocol to avoid interobserver bias.

### Trial medication

All subjects received four tablets of SET or placebo of identical shape and colour three times a day starting 72 hours before exhaustive eccentric exercise day and for 72 hours post exercise. Trial medication was taken on an empty stomach 30 min before meals with 250 mL water. Date and time of intake and time of subsequent meals were documented in a diary. Residual tablets were counted to control for compliance.

### Outcome measures

The maximal concentric strength of the *M. Quadriceps femoris* of both legs was assessed at screening (2–4 weeks before visit 1), pre visit 1 (day 0, before start of trial medication), at visit 1 (day 4 of administration of trial medication), immediately before (pre) and immediately after exhaustive eccentric exercise (0 hour), as well as after 3 hours and 6 hours, and at day 5 (24 hours), day 6 (48 hours) and day 7 (72 hours). PTM and angle of peak torque were measured as parameters of maximal concentric strength.

Pressure-induced pain (PIP) was assessed by the same observer for all subjects using a 1 cm^2^ metal disk against the middle of the muscle belly. Pressure was constantly increased until it became unpleasant. PIP was assessed at the indicated time points and the mean of three tests was used for analysis.

### Biomarkers

Biomarkers of muscle metabolism and damage, inflammatory and immune response, and redox status were determined. Samples were taken at individual time points, stored frozen below −80°C, except for lactate and natural killer (NK) cell activity, and analysed batchwise for each subject after trial completion according to manufacturer’s instructions, if not otherwise indicated. Creatine kinase and lactate dehydrogenase as markers of muscle damage were determined at Medizinisches Versorgungszentrum Leinfelden for samples taken pre-exercise, and at 0, 3, 6, 24, 48 and 72 hours after exercise. Lactate was determined pre-exercise, and 10 and 30 min after exercise taken by capillary blood samples by trial site. Inflammatory response was investigated as IL-6 (pre-exercise, and 0, 3 and 24 hours after exercise) (Quantikine High Sensitivity ELISA; R&D Systems) and prostaglandin E derived from cyclooxygenase 2 (pre-exercise, and 3, 6 and 24 hours) (Prostaglandin E Metabolite EIA kit; Cayman Chemical) at BioTeSys, Esslingen. Immune function was investigated by IL-2 inducible NK cell activity (pre, 3 hours, 24 hours) at the Institut für Medizinische Diagnostik, Berlin.^37 38^ Total antioxidative status (TAS) and total oxidative status (TOS) (pre, 3 hours, 6 hours, 24 hours) were assessed using a photometric test assay (ImAnOx [TAS/TAC] Kit; perOS [TOS/TOC] Kit; Immundiagnostik AG, Bensheim, Germany).

### Safety assessments

Blood samples for routine laboratory tests, including haemogram, liver enzymes, lipids, glucose, uric acid and creatinine, were taken at screening, before, and 24 and 72 hours after exercise and analysed by Synlab Medizinisches Versorgungszentrum Leinfelden. Vital signs were determined at all visits. Concomitant medication and adverse events were documented.

### Statistical analysis

As no single measure captures the multidimensional nature of recovery from EIMD, the combination of single efficacy endpoints *reduction of maximal strength* (*PTM*) and *PIP* was chosen to be evaluated by a multivariate, directional test approach in stage I of the trial.^39^

The multiple level *alpha* of the trial (multiple level of significance) was defined as α=0.025 (one sided). The confirmatory analyses were performed with the intention-to-treat population. Missing values were replaced by last observation carried forward technique. Results are given as p values and effect size measures with their CIs (Mann-Whitney (MW) statistic as corresponding effect size measure of the Wilcoxon-Mann-Whitney test). The traditional benchmarks for the MW effect size measure are as follows: 0.29 = large inferiority, 0.36 = medium inferiority, 0.44 = small inferiority, 0.50 = equality, 0.56 = small superiority, 0.64 = medium superiority and 0.71 = large superiority.

Multiple a priori ordered hypothesis testing was performed as a two-stage procedure^30 40 41^ including the possibility to stop the trial after stage I due to success (proof of efficacy) (p_1_≤α_1_=0.0102) and futility (p_1_≥α_0_=0.5) or to plan stage II of the trial based on results of stage I, including reassessment of sample size and finalisation of the trial design. This procedure based on Fisher’s combination test shows only negligible loss in test power compared with fixed sample size trials^42^ and uses the adjusted value α_1_=0.0102 as a critical value for the test of stage I.^34^ Recalculated total sample size for stage II was 2×22 subjects with no crossover design.

## Results

### Trial population

**Figure 1:**
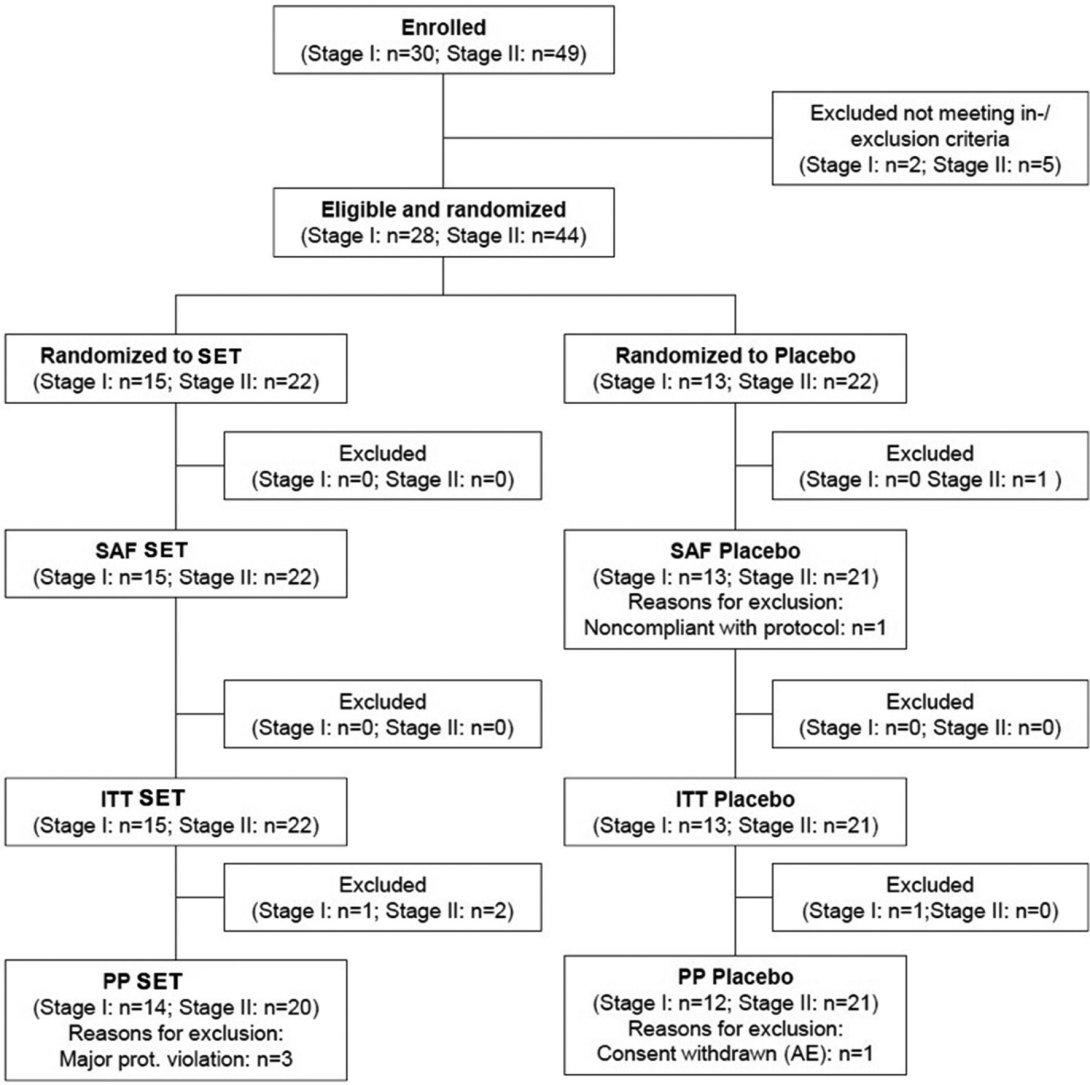
Disposition of subjects to stages I and II and different analysis populations. AE, adverse event; ITT, intent-to-treat population; n, number; PP, per protocol population; SAF, safety population; SET, systemic enzyme therapy (Wobenzym).

In stage I, 30 subjects were enrolled between 20 April 20 and 1 August 2013, and 28 subjects (safety population) were treated in a crossover design. Per protocol (PP) analysis consisted of 26 subjects (figure 1).

In stage II, a total of 44 subjects were enrolled between 15 May and 27 July 2014. The safety population was comprised of all enrolled and treated subjects (n=44). The PP population (41 subjects; SET: 20; placebo: 21) resulted from exclusion of two subjects due to protocol violation (timing of PTM >20% and severe violation of screening PTM) (figure 1).

### Stage I

**Figure 2:**
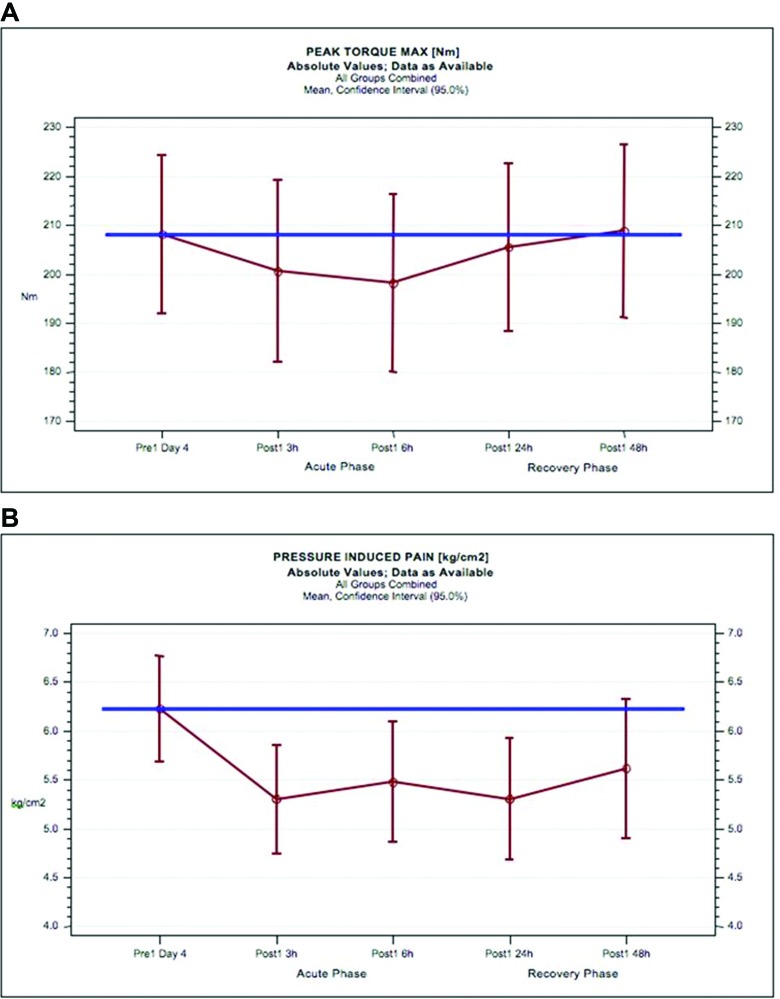
Stage I results for peak torque maximum and pressure-induced pain in response to exhaustive eccentric exercise in all subjects. Mean values of peak torque maximum (Nm) (A) and pressure-induced pain (kg/cm^2^) (B) and 95% CIs for all subjects of the total blinded review population of stage I (red circles and lines) compared with the mean baseline peak torque maximum of all subjects (blue line).

The PTM and PIP results for both treatment groups (all subjects pooled) compared with mean baseline values are provided in figure 2.

As expected, PTM is reduced in the ‘acute’ phase at 3 hours (3.4%; 201 Nm) and 6 hours (4.8%; 198 Nm) after exhaustive eccentric exercise compared with baseline (100%; 208 Nm) (figure 2A). However, during'recovery' phase (24–48 hours), the average PTM returns to 205 Nm at 24 hours and to 209 Nm at 48 hours, the level before exercise. Thus, the potential to discover differences between two treatments is substantially reduced beyond 24 hours. PIP measured by algometry (both treatment groups combined) resulted in a reduction of pain threshold after exhaustive eccentric exercise in both phases (figure 2B).

**Figure 3:**
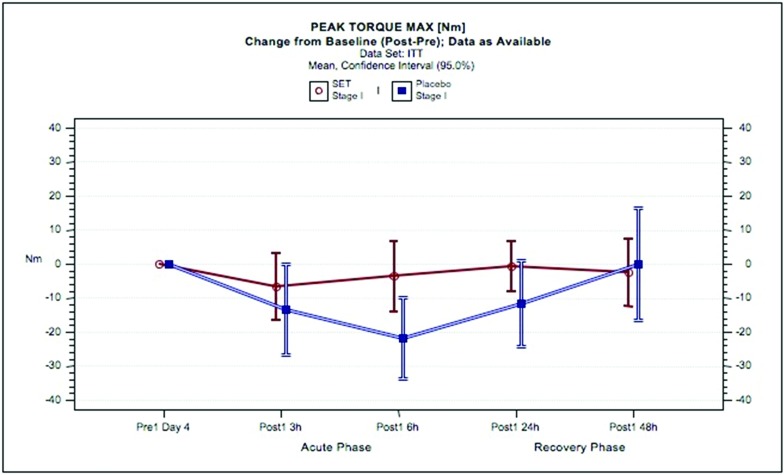
Peak torque maximum in response to exhaustive eccentric exercise in subjects administered SET or placebo. Mean changes from baseline of peak torque maximum [Nm] and 95% CIs for the intent-to-treat population are shown for SET (red circles and lines) and for placebo (blue circles and lines). ITT, intent-to-treat population; SET, systemic enzyme therapy.

The PTM results in stage I (phase 1) with SET or placebo are shown in figure 3. Exhaustive eccentric exercise led to a reduction in PTM in the SET group of 2.8% at 3 hours and 1.5% at 6 hours compared with placebo of 6.2%, 10% and 5.7% at 3, 6 and 24 hours, respectively. Physical performance returned to baseline at 24 hours with SET but not until 48 hours with placebo. The associated MW effect size indicated more than ‘small’ superiority of the SET group (MW=0.6153, p=0.0332) compared with placebo.

### Stage II

**Figure 4:**
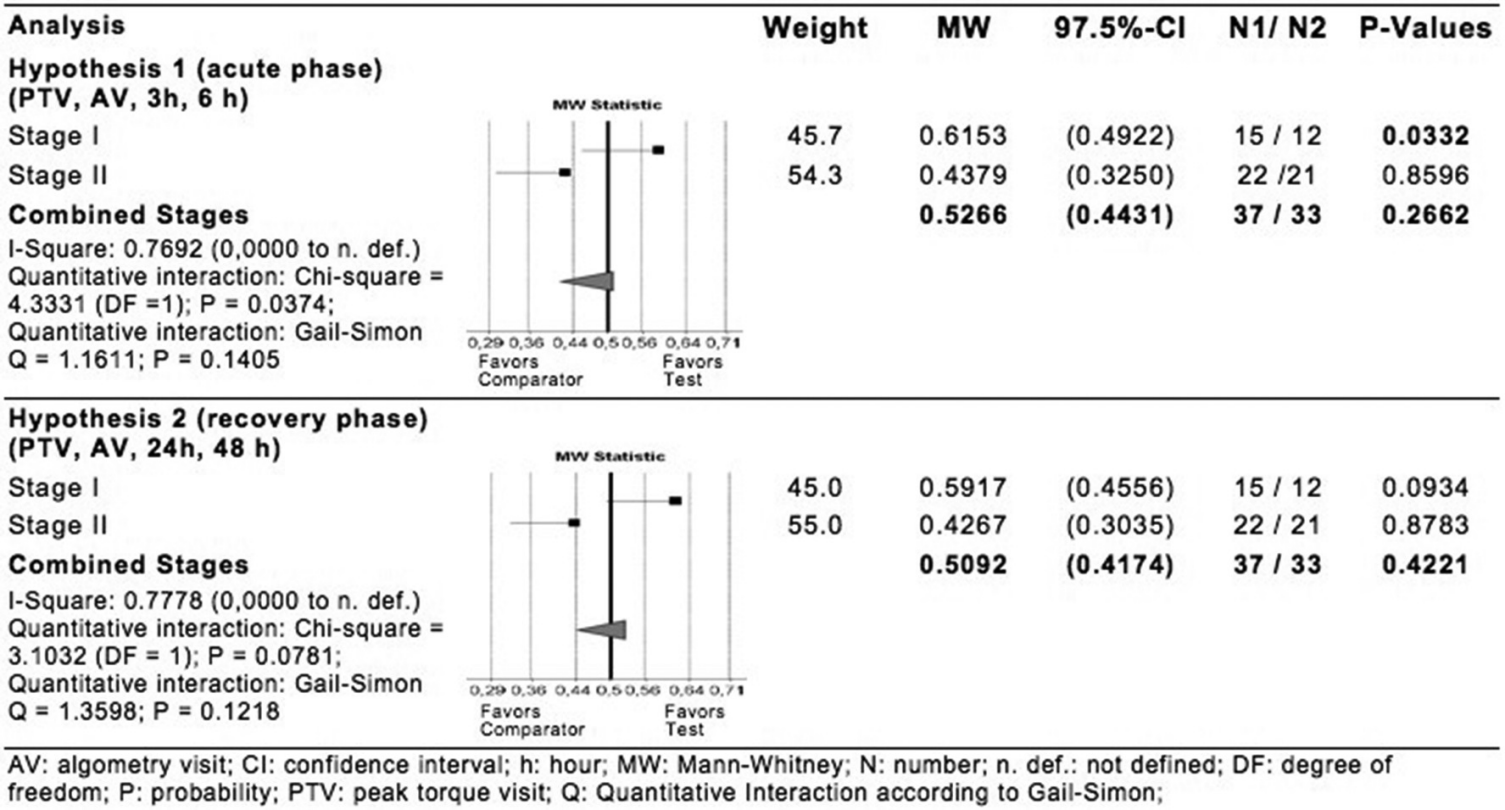
Statistical analysis of multidimensional ensemble of peak torque maximum and pressure-induced pain at 3 hours and 6 hours (hypothesis 1) and at 24 hours and 48 hours (hypothesis 2).

In contrast to stage I, confirmatory analysis of both hypotheses of stage II resulted in no evidence for corresponding treatment effects (hypothesis 1: p=0.8596, hypothesis 2: p=0.8783, both one sided). As shown in figure 4, there is ‘severe’ heterogeneity between both stages for both hypotheses (hypothesis 1: MW=0.6153 vs MW=0.4379; I^2^=0.7692, p=0.0374; hypothesis 2: MW=0.5917 vs MW=0.4267; I^2^=0.6778, p=0.0781). As both I^2^ values are above 0.5, indicating ‘large’ heterogeneity, results are to be interpreted separately for each stage.

### Biomarkers

**Figure 5:**
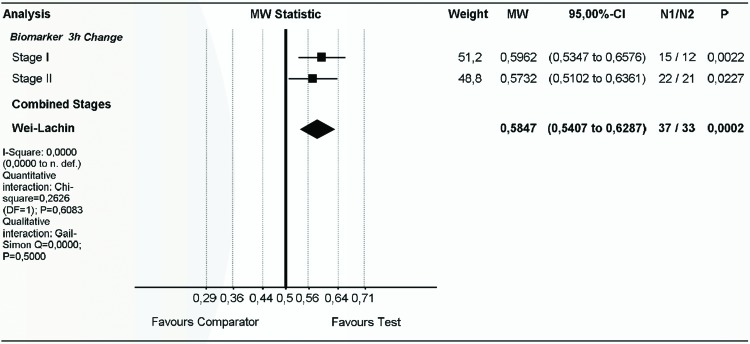
Superiority of SET compared with placebo in improving inflammatory, immune and metabolic biomarkers in response to exhaustive eccentric exercise. Effect sizes (changes from baseline) at 3 hours after exhaustive eccentric exercise were pooled for stage I, stage II and both stages combined. Pooled biomarkers include creatine kinase, lactate dehydrogenase, lactate, interleukin 6, prostaglandin E2, total oxidative status, total antioxidative status and natural killer cells, and were analysed for the direction of superiority. df, degrees of freedom; MW, Mann-Whitney; N, number; n. def., not defined; p, probability; SET, systemic enzyme therapy.

Pooled biomarker analysis at 3 hours after exercise demonstrated significant advantages for SET compared with placebo (stage I: p=0.0011; stage II: p=0.0114) (figure 5) and no heterogeneity (I^2^=0.0) between stages I and II. Stage I and II combined biomarker effect size shows significant and ‘more than small’ superiority of the SET group (MW=0.5847, p=0.0001) compared with the placebo group (figure 5).

### Safety 

In stage I, out of 16 single adverse events, only one was characterised as ‘possibly’ related (diarrhoea, SET). In stage II, two single adverse events, one possibly related to SET (acne-like rush at chin and mild pruritus), were reported. No adverse event was ‘serious.’ Both events related to SET were characterised as mild and resolved. In both stages, there were no clinically relevant findings in any of the treatment groups. SET is well tolerated at the dosage of 3×4 tablets per day.

## Discussion

We demonstrated the superiority of SET compared with placebo in maintaining strength and reducing pain in response to exhaustive eccentric exercise (acute phase, stage I). Stage II data were evaluated separately due to significant heterogeneity (I^2^>0.5) and, in contrast to stage I, did not result in differences of SET or placebo on EIMD. Biomarkers responded to SET across both study stages.

### Strengths and limitations

The trial was carefully planned using a multidimensional approach for outcome assessment and classification, taking into account the multidimensional aspect of recovery from EIMD. Additionally, a two-stage approach with interim analysis and continuation with refined hypotheses and readjusted sample size offered all opportunities to finalise the trial successfully.

Surprisingly, the preplanned test for carryover effects was statistically significant on the defined level α_1_ (p=0.0092, one sided, Wei-Lachin procedure, Bauer-Köhne α_1_=0.0102) in stage I. Thus, according to statistical plan, only phase 1 data from stage I could be used for non-parametric confirmatory analysis, and the crossover approach had to be abandoned in stage II in favour of a parallel group comparison. One possible explanation for carryover effects is that impairment of muscle function could have been influenced by recent, that is, within some months,^43^ high-force eccentric work using the same muscle(s). This effect is commonly referred to as the ‘repeated-bout effect.’^44^ However, as EIMD during the current trial was only mild, the washout phase during the eccentric exercise sessions should have been sufficient to avoid a repeated bout effect. A carryover effect from SET to placebo cannot be excluded as irreversible changes of cell surface receptors of blood and immune cells have been reported.^20 21 23^

Stages I and II revealed ‘severe’ heterogeneity even though inclusion criteria were unchanged at screening for both stages at PTM 150–300 Nm and a variability of below 20%. Large interindividual variation in response to eccentric exercise is commonly reported.^45^ EIMD should even be higher in this age range as the magnitude of muscle damage is increased from preadolescent, adolescent to postadolescent men.^46^ Flexibility,^47 48^ eccentric peak and end-range torque^49^ as well as angle of peak torque (cf. the joint angle–torque relationship) are further factors contributing generally to a variety of EIMD responses.^50^ Additionally, extended training of the same muscle(s) comparable to exhaustive eccentric exercise may influence regeneration mechanisms and individual autonomous pain threshold.^45^ Large variations in individual responses to eccentric exercise are also evident from the literature, and gross muscle damage does not occur in all individuals.^45 51–54^

Study model may be limited in ability to observe a functional response in more strength-trained athletes

A closer look at single subject data at baseline revealed differences between stages in the number of main disciplines and training duration per week (table 1).

**Table 1:** Summary of anthropometric data and baseline characteristics

Parameter	Stage I			Stage II		
	Total (n=27)	SET (n=15)	Placebo (n=12)	Total (n=42)	SET (n=21)	Placebo (n=21)
Age (years)±SD	31.6±9.3	29.2±8.8	34.6±9.3	29.7±8.7	30.7±7.6	28.7±9.8
Height (m)±SD	1.81±0.06	1.79±0.06	1.83±0.07	1.83±0.05	1.84±0.06	1.82±0.04
Weight (kg)±SD	80.0±10,0	76.5±10.2	84.3±8.0	81.2±7.4	81.0±8.6	81.3±61
BMI (kg/m^2^)±SD	24.4±2.1	23.8±2.0	25.2±1.9	24.3±1.8	24.0±1.8	24.6±1.8
Sport activity (min/week)±SD	369±206	415±197	312±211	482±433	483±502	481±363
CK (U/L)±SD	214±157	227±148	199±122	244±165	225±160	264±172
PTM/BW (Nm/kg)±SD	2.76±0.44	2.76±0.51	2.75±0.34	2.82±0.51	2.75±0,53	2.89±0.48

Anthropometric data, sporting activity and creatine kinase were assessed at screening; peak torque maximum/body weight was measured the day before start of supplementation.

BMI, body mass index; CK, creatine kinase; Nm, newton metre; PTM/BW, peak torque maximum/body weight; SET, systemic enzyme therapy.

Participants in stage I were primarily runners/joggers (endurance training) whereas participants in stage II primarily focused on strength (resistance) training. The duration of weekly strength training was reported at nearly three times for stage II compared with stage I (179 vs 61 min). Further, overall training duration across all disciplines was higher in stage II than stage I (482 vs 369 min). Therefore, stage II participants were more well trained than stage I participants, particularly related to strength (resistance) exercises. This may increase PTM by higher physical performance and by increase in pain threshold or the ability to increase PTM despite pain. Insensitivity of the study model to detect advantages of SET might have been caused by these differences in baseline resistance training. It has been suggested to report individual data and to classify subjects, for example, low, medium/moderate or high responders, to allow for a better presentation and interpretation of the data.^49 51^

### Biomarker data confirm anti-inflammatory effects of SET

In contrast to functional parameters PTM and PIP, biomarker analysis resulted in a significant and ‘more than small’ superiority of the SET group compared with placebo group in single stages and both stages combined. Cytokines may play a relatively minor role in regulating the health benefits of low-intensity exercise, such as brisk walking.^55^ In contrast, marathon running induces high physiological stress and a large cytokine response^56^ as a more generalised response to internal and/or external stress. Factors such as oxidative or nitrosative stress, damaged or unfolded proteins, hyperthermia or energy imbalance likely induce cytokine production during exercise through catecholamines, endotoxin, alarmins, ATP and proinflammatory cytokines themselves.^57 58^ SET is likely to contribute to a reduction of large cytokine response in EIMD and DOMS independent of physical performance/training status.

## Conclusion

SET administered orally 72 hours before and 72 hours post exhaustive eccentric exercise resulted in higher maximal concentric strength and lower PIP in subjects who were less experienced in resistance training and in significant favourable effects on anti-inflammatory and other biomarkers in all subjects. The potential role of SET in managing EIMD and DOMS across different training groups needs further investigation.

## Footnotes

i Wobenzym plus contains 67, 5–76, 5 mg bromelain (standardised to 450 FlP units); 32–48 mg trypsin (standardised to 24 μkat) and 100 mg rutoside 3 H2O, and is manufactured by Mucos Pharma GmbH & Co. KG, Berlin.
